# Back beliefs among elderly seeking health care due to back pain; psychometric properties of the Norwegian version of the back beliefs questionnaire

**DOI:** 10.1186/s12891-019-2910-8

**Published:** 2019-11-03

**Authors:** Alexander Tingulstad, Rikke Munk, Margreth Grotle, Ørjan Vigdal, Kjersti Storheim, Birgitta Langhammer

**Affiliations:** 1 Oslo Metropolitan University, Pilestredet 44, 0167 Oslo, Norway; 2 Research and Communication Unit for Musculoskeletal Health, Oslo University Hospital, P.B. 4950, Nydalen, 0424 Oslo, Norway; 3 Sunnaas HF, Bjørnemyrveien 11, 1453 Bjørnemyr, Norway

**Keywords:** Back beliefs questionnaire, BBQ, Back pain, Validity, Reliability, Elderly

## Abstract

**Background:**

The Back Beliefs Questionnaire (BBQ) is a 14-item patient-reported questionnaire that measures attitudes and beliefs about the consequences of back pain. The BBQ has recently been translated into Norwegian, but its psychometric properties have not yet been tested. The aim of this study is to evaluate the reliability and construct validity of the BBQ when used on elderly patients with back pain.

**Method:**

A prospective cohort study with a test-retest design among 116 elderly patients (> 55 years of age) seeking primary care for a new episode of back pain. Test-retest, standard error of measurement (SEM), minimal detectable change (MDC), internal consistency and construct validity by a priori hypotheses (Spearman’s- and Pearson correlation coefficient) were tested.

**Results:**

A total of 116 patients, mean age (SD) 67.7 (8.3), were included and 63 patients responded to the test-retest assessment. The mean (SD) BBQ sum scores (range 9–45) were 29.8 (7.0) and 29.2 (6.7) for the test and retest respectively. The test-retest was acceptable with an intraclass correlation coefficient of 0.71 (95% CI, 0.54–0.82), SEM was 3.8 and MDC 10.5. Internal consistency with Cronbach’s alpha was good (0.82) and acceptable construct validity was supported by the confirmation of 75% of the a priori hypotheses.

**Conclusion:**

The Norwegian version of the BBQ demonstrated acceptable test-retest reliability and good construct validity and can be used to assess pessimistic beliefs in elderly patients with back pain.

## Introduction

Back pain is among the most common musculoskeletal complaints seen in primary care [1]. The prevalence of back pain has been rising continuously for many years and the financial burden on society is increasing [1–3]. Although our population is aging globally, the elderly are often excluded from research on back pain and the influence of psychological factors [4]. Psychological factors, in particular beliefs about back pain, have been shown to play a major role for the course of back pain [5–8]. Negative and irrational beliefs are associated with persistent back pain [5].

Beliefs and attitudes towards back pain can be measured with questionnaires. The Back Beliefs Questionnaire (BBQ) was developed by Symonds et al., with the aim to make a new instrument to measure beliefs and attitudes related to back pain [5]. The authors developed a 14-item self-report questionnaire to investigate beliefs about the inevitable consequences of back pain [5]. BBQ has been used to predict recovery rate from back pain [9, 10], in population studies assessing public attitudes and as an outcome to assess effectiveness of educational campaigns [11–13]. To our knowledge, the BBQ has been translated into Arabic [14, 15], German [10], Chinese [16, 17] and French [18]. Most of these methodological studies have shown good test-retest reliability and validity. However, if BBQ is to be used as a measurement outcome in addition to a predictor, test-retest reliability in terms of minimal detectable change (MDC), standard error of measurement (SEM) and limits of agreement (LOA) needs to be established. These estimates are useful as they provide an interpretation of measurement error according to the absolute score of an instrument [19], however, few studies have investigated these properties [14, 15].

The BBQ has recently been translated into Norwegian, but assessment of test-retest reliability and validity has not yet been performed. The psychometric properties of any scale may be affected by translation into another language, hence, it is important for the scale to be evaluated psychometrically. Therefore, the aim of this study was to evaluate the psychometric properties of the Norwegian version of the BBQ in terms of test-retest reliability, construct validity and internal consistency when used on elderly patients with a new episode of back pain.

## Material and methods

This methodological study is part of the BACE (BACk Complaints in Elders) study in Norway. BACE is a prospective cohort study designed to assess elderly (> 55 years) patients with back pain. The protocol has been published [20].

### Translation and cross-cultural adaption

The English version of the BBQ was translated and cross-culturally adapted into Norwegian according to guidelines [21]. Two translators (one philologist and one clinician), whose mother tongue is Norwegian, independently translated the BBQ into Norwegian, and synthesized them into one Norwegian version. Two native English speakers, blinded to the original BBQ, independently performed the back translation and synthesized the two versions into one English version. An expert committee consisting of the translators and two researchers from our research group (MG and RM) reviewed the translations and agreed on a prefinal version. The prefinal version was tested on ten participants at baseline with similar characteristics as the whole sample. The items were confirmed to be relevant and understandable without any proposed alterations. Hence, the final version of the Norwegian BBQ evaluated in this study is the same as the prefinal version.

### Participants

Eligible participants were patients aged 55 years or older who had attended a consultation with a primary care practitioner regarding a new episode of back pain. Back pain was defined as pain from the cranial ridge of the scapula to S1. Patients were excluded if they had difficulty with completing the questionnaire due to language barriers or if they had received treatment for the same episode of back pain within the last 6 months. All patients received written and oral information about the study and informed consent was signed by all participants.

### Procedures and measurements

The BBQ was administered to all participants as part of a comprehensive questionnaire used in the cohort. This included sociodemographic variables (age, gender, education, work status), medical history and several questionnaires [20]. The questionnaires were self-administered by all patients, using a tablet computer, alone in a separate room. Baseline (T0) also consisted of a clinical examination of the patients [20]. Test-retest reliability was assessed by testing the BBQ on a sub-sample of the patients. At baseline, patients were asked to fill out the retest (T1) at home, until a sufficient number (> 50) of participants had completed the retest. The participants were asked to fill out the second questionnaire 2 days after the baseline testing.

The BBQ is a patient-reported questionnaire consisting of 14 statements regarding beliefs about the consequences of back pain, with items such as “Back trouble will eventually stop you from working” and “Back trouble makes everything in life worse”. Each item is scored on a 5-point Likert scale ranging from strongly disagree (1) to strongly agree (5). Five of the items are distractors and the remaining 9 items are used in the scoring of the questionnaire, resulting in a score ranging from 9 to 45. The scores are reversed before they are summarized, meaning that a low score indicates more pessimistic beliefs regarding the consequences of back pain [5]. The BBQ instrument is shown in the Additional file 1.

Several reference scales were used to evaluate convergent and divergent construct validity. For convergent validity we used, the Fear-Avoidance Beliefs Questionnaire for physical activity (FABQ-PA) [6], the Roland-Morris Disability Questionnaire (RMDQ) [22] and the Pain Catastrophizing Scale (PCS) [23], for divergent validity we used the Numeric Rating Scale (NRS). FABQ-PA consists of 5 statements that evaluates fear and avoidance behavior introduced through physical activity [6]. The questionnaire has been translated into Norwegian and has shown acceptable psychometric properties in Norwegian patients with low back pain [24]. Acceptable results were also obtained when assessing the reliability and validity of a Norwegian version of the RMDQ [25]. This questionnaire examines functional status related to normal activities of daily life [22]. PCS includes 13 items that focus on thoughts and feelings about pain [23]. A Norwegian version tested on patients with back pain has demonstrated acceptable psychometric properties [26]. NRS has been widely used to evaluate pain and has proven to be preferable when examining low back pain patients [27].

### Statistical analysis/analysis

IBM SPSS Statistics for Windows, version 23.0 (IBM Corp, Armonk, NY) was used for all data analyses. Descriptive analyses included means and standard deviation (SD) for numerical variables or frequencies for categorical variables. The sample size was based on the quality criteria proposed by Terwee et al. [19]. This suggested criteria recommends a minimum of 50 participants when exploring test-retest reliability, and at least 100 participants when exploring internal consistency and construct validity. Kolmogorov-Smirnov tests for normality and the visual inspection of distribution plots were used to determine the distribution of the BBQ-scores.

Floor and ceiling effects were assessed by evaluating the numbers of participants with the lowest or highest score. They were considered to be present if more than 15% of the participants had the lowest or the highest possible score [19]. In addition, data quality was assessed by evaluating missing data.

Test-retest reliability was assessed by calculating the intraclass correlation coefficient (ICC) from a two-way random effects model, absolute agreement (2,1)**.** Since each subject was only measured once at the test and the retest, the single measure value is used in the results. A minimum ICC of 0.7 was considered acceptable [19]. Measurement error was demonstrated by the standard error of measurement (SEM) and minimal detectable change (MDC). The formula for SEM is SEM = SD√1 – ICC and MDC are calculated with MDC = 1.96 × √2 × SEM. Bland-Altman plots were constructed to assess how much the scores can vary in stable persons throughout the scale, with the agreement of the test-retest of the BBQ. Limits of agreement (95%) were calculated with the formula [mean difference ± 1.96 × SD_difference_] [28].

The internal consistency of the questionnaire was assessed with Cronbach’s Alpha. Values ranging from 0 to 1 are considered good when above 0.8, moderate between 0.7 and 0.8, and low when under 0.7 [29].

Construct validity for the BBQ total score was assessed by comparing the BBQ for association to concurrent measures. Predefined hypotheses of association were established based on the construct of the measures and former correlations in similar studies. The BBQ was hypothesized to have moderate to high correlation with the FABQ-PA, moderate correlation with the RMDQ, high correlation with the PCS, and low to moderate correlation with the NRS. A high score in reference scales indicates more fear avoidance, catastrophizing, disability and pain, and a low score in BBQ reflects pessimistic beliefs and attitudes, meaning that all correlations were expected to have negative values. The Pearson’s correlation coefficient was used if values were normally distributed and the Spearman’s correlation coefficient if values were not normally distributed. Correlation between scales was interpreted as high when r was 0.50 and above, moderate when between 0.30 and 0.49, and low when between 0.10 and 0.29 [30]. An acceptable construct validity of the BBQ was obtained if at least 75% of the hypotheses were confirmed [19].

## Results

### Patients and data quality

The prefinal version of the Norwegian BBQ was tested on the first ten participants at baseline, with similar characteristics as the whole sample. The ten participants included 6 women and 4 men, with a mean age of 69.1, all with a history of back pain and a mean (SD) BBQ score of 27.6 (4.9). The main study included 116 patients from primary care with back pain in the validation (T0), 71 women and 45 men. Of those included, 10 patients had missing items in the BBQ and were excluded from the analysis, resulting in a total of 106 participants with valid data. Sixty-three participants had valid data at retest (T1). The 10 incomplete responses had a total of 25 missing items, within which every item on the scale was represented. Items 13 (“Back trouble must be rested”) and 14 (“Later in life back trouble gets progressively worse”) had five missing values each, and the other items each had 1–3 missing values. Among the distractors, item 7 had four missing values, and item 4,5,9 and 11 had 1–2 missing values. The mean (SD) BBQ total score at T0 was 29.8 (7.03). The lowest (9) and highest (45) possible scores were each achieved by one participant, indicating no floor- or ceiling effects for the BBQ total score. Patient characteristics and clinical variables are presented in Table 1.

**Table 1:** Characteristics of patients in the whole sample (T0) (*n* = 116) and the test-retest sub-group (T1) (*n* = 63)

Age (yrs.)	116	67.7 (8.32)	63	68.6 (8.69)
Sex (% Women)	116	71 (61)	63	38 (60)
Civil status (%)	115		62	
Married/cohabiting		85 (73)		47 (76)
Single		19 (16)		8 (13)
Widow/widower		11 (10)		7 (11)
Language (%)	114		61	
Norwegian		109 (94)		59 (97)
Other		5 (4)		2 (3)
Education (< 12 years) (%)	113	62 (53)	61	29 (48)
Pain medication (yes) (%)	115	50 (43)	62	25 (40)
Employed (%)	95	35 (37)	48	17 (35)
Smoking (yes) (%)	115	10 (9)	62	3 (5)
Expectations about back pain in 3 months (%)	114		63	
Healed		21 (18)		12 (19)
Much better		68 (59)		39 (62)
No change		24 (21)		11 (18)
Much worse		1 (1)		1 (2)
Worse than ever		0 (0)		0 (0)
History of back pain (yes) (%)	114	111 (97)	62	61 (98)
Frequency of back pain (%)	109		60	
Monthly		42 (39)		24 (40)
Yearly		36 (33)		21 (35)
Less than once per year		19 (17)		10 (17)
Once every 5 years		9 (8)		4 (7)
One time		3 (3)		1 (2)
BBQ (9–45), mean (SD)	106	29.8 (7.03)	57	29.2 (6.74)
NRS (0–10), median (range)	110	6 (0–10)	61	5.4 (0–10)
FABQ-PA (0–24), mean (SD)	116	10.1 (6.08)	63	10.1 (6.78)
PCS (0–52), median (range)	116	12 (0–48)	63	11.8 (0–39)
RMDQ (0–24). mean (SD)	116	10 (5.13)	63	9.8 (4.79)

*BBQ* Back Beliefs Questionnaire; *NRS* Numeric Rating Scale; *FABQ* Fear-Avoidance Beliefs Questionnaire; *RMDQ* Roland-Morris Disability Questionnaire; *PCS* Pain Catastrophizing Scale

### Reproducibility

**Fig. 1:**
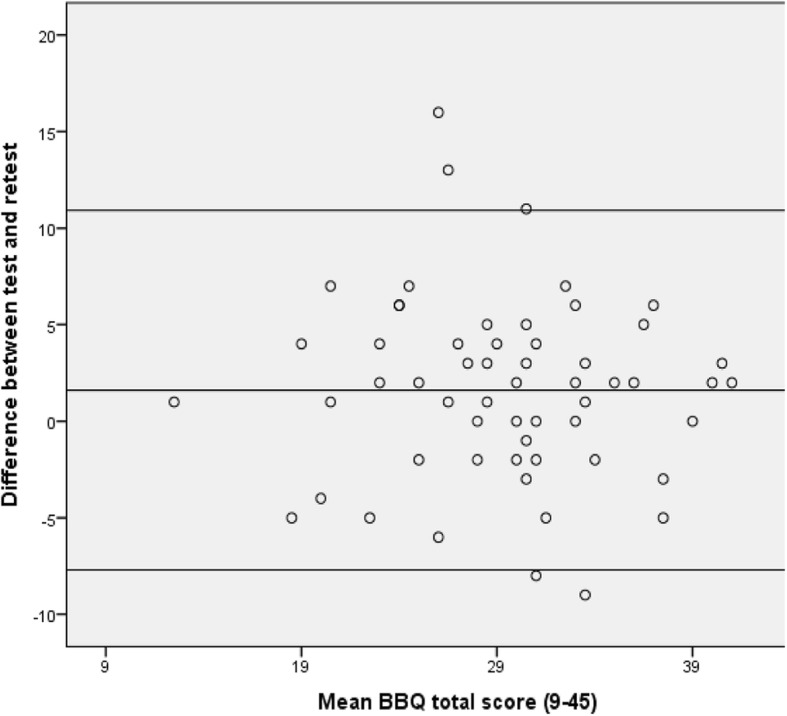
Bland-Altman plot of mean difference between test and retest of the Back Beliefs Questionnaire (*n* = 57) and the Limits of Agreement

The median time between test and retest was 3 days (range 1–13). There was an acceptable agreement between the test and retest total score, with an ICC (2,1) of 0.71 (95% CI, 0.54–0.82). The SEM of the BBQ total score was 3.8, the MDC was 10.5 and MDC% was 23.3. The Bland-Altman plot (Fig. 1) demonstrates a mean difference between test and retest of 1.6 points, and limits of agreement of 10.9 and − 7.7 points of the total score from 9 to 45. The internal consistency, assessed by Cronbach’s alpha, was 0.82 for the BBQ at T0. At T1, BBQ demonstrated a Cronbach’s alpha value of 0.80.

### Construct validity

Evaluation of normality with Kolmogorov-Smirnov tests and the visual inspection of distribution plots suggested that BBQ, FABQ-PA and RMDQ, were normally distributed, while PCS and NRS were non-normally distributed. Table 2 presents the predefined hypotheses and the correlation analyses between BBQ and FABQ-PA, PCS, RMDQ and NRS. Reversal of the BBQ score leads to negative correlation coefficients. The BBQ total score showed a strong correlation coefficient to the FABQ-PA (*r* = − 0.57) and, moderate correlation to both PCS (rho = − 0.45) and RMDQ (*r* = − 0.49). Moreover, the BBQ demonstrated a weak correlation to the NRS (rho = − 0.14). In total, the correlation coefficients confirmed 75% of the a priori hypotheses.

**Table 2:** Construct validity: a priori formulated hypothesis

Hypothesis	Correlation value	N	Hypothesis confirmed?
The BBQ was expected to have moderate to high correlation with FABQ-PA	−.573^a^	106	Yes
The BBQ was expected to have moderate correlation with RMDQ	−.494^a^	106	Yes
The BBQ was expected to have high correlation with PCS	−.447^a^	106	No
The BBQ was expected to have low to moderate correlation with NRS	−.138^a^	101	Yes

^a^ Significant correlation (< 0.01). *BBQ* Back Beliefs Questionnaire; *NRS* Numeric Rating Scale; *FABQ* Fear-Avoidance Beliefs Questionnaire; *RMDQ* Roland-Morris Disability Questionnaire; *PCS* Pain Catastrophizing Scale

## Discussion

The Norwegian version of the BBQ shows acceptable psychometric properties in elderly patients with a new episode of back pain. Our results indicate that BBQ can be used in both clinical settings and research with the purpose of assessing beliefs about back pain. This is in line with former assessments of BBQ in other languages [14, 15, 31]. Our study is the first report to evaluate psychometric properties in the Norwegian version of the BBQ. Additionally, the fact that our research was conducted in elderly patients with back pain contributes important knowledge to a field in which most research has been conducted in younger populations [4, 32].

Fears and beliefs leading to avoidance have been shown to negatively influence the prognosis of back pain and increases the risk of developing chronic disability [9, 33]. With BBQ as an examination measurement, it is possible to detect negative beliefs in patients with back pain. Early detection will allow primary healthcare workers to provide back pain patients with clarifying information pertaining to their irrational beliefs. This positive influence may have an important socioeconomic impact worldwide.

Our study sample completed the BBQ with a mean score of approximately 30 on a scale ranging from 9 to 45; this is a relatively high score, reflecting optimistic beliefs. This score is higher than some other studies which report a mean score ranging from 21 to 26 [14, 15, 18, 34], but similar to a study from Australia which reports a mean BBQ score of 30.7 [33]. The low level of negative beliefs in our sample of elderly people might have been influenced by different coping strategies, reduced pain perception and it might be argued that some elderly patients believe pain to be a normal part of the ageing process and have more realistic beliefs [35, 36]. Only 35% of our participants were working, and one could speculate that retired participants may experience fewer consequences due to an episode of back pain as they are unconcerned by the responsibilities of employment and with taking sick leave. Since previous studies have been conducted on different populations, such as healthcare workers [17], healthcare students [16], healthy individuals [11], and younger patients [18] as well as in different cultures [14, 15, 34], it is difficult to make any direct comparisons with this study. Furthermore, back beliefs can change rapidly, which can influence evaluations using the BBQ. While we found a low correlation between the BBQ and pain, in their research, Bostick and associates found that BBQ participants achieved lower scores when experiencing acute and severe pain, while a 1-week history of mild back pain resulted in higher scores [31]. The ICC was considered to be acceptable (0.71) according to our chosen classification, which suggests that the BBQ is a reliable outcome measure in our population [19]. In earlier studies, the BBQ has demonstrated ICC with results ranging from 0.80–0.89 [10, 14–17]. Few authors specify the chosen effects model and measure regarding the ICC, which can influence the outcome results. Measuring back beliefs with retest might be challenging. There is a potential risk of recall bias if the participant has a short interval between the test and the retest, and a risk of possible change in back pain status when the time between tests is long. A short interval was chosen in this study, since the high number of questionnaires completed at T0 would most likely reduce recall bias of BBQ at T1 2 days later**.** The ICC is influenced by the variation between the patients – heterogeneity resulting in a high ICC value – and substantiates the importance of assessing measurement errors [37]. MDC, which expresses an error estimate given in the scale’s unit, resulted in 10.5 of a possible 45 points. MDC determines the smallest within-person change to ensure that the change is larger than the measurement error, and 10.5 points will provide an estimate of this limit when using the BBQ as an outcome measure. The results (Fig. 1) from LOA show that the large measurement error was equally spread across the whole scale range. This implies that when using the BBQ as an outcome to evaluate change during a treatment or clinical course, an observed change below 10.5 points can not be distinguished from measurement error, regardless of baseline value. This estimate of measurement error is large taking into consideration the scale from 0 to 45. To the authors’ knowledge, few studies have investigated the measurement error of the BBQ. Alamrani and coworkers obtained a somewhat lower MDC (5.9) and SEM (2.1) values influenced by their high ICC value (0.88) [15]. Due to the high measurement error in this study, more reports should investigate of measurement errors for the BBQ, which may increase our confidence in utilizing the questionnaire as an outcome measure.

A high internal consistency was found for BBQ, even though the Cronbach’s alpha coefficient is sensitive to the number of items in the scale, and questionnaires with fewer than 10 items can result in a value that is too low [38, 39]. The results are consistent with other studies conducted on BBQ, although our values of 0.82 (test) and 0.80 (retest) are slightly higher than in most previous studies. Previous publications have demonstrated Cronbach’s alphas ranging from 0.70 to 0.80 [5, 10, 14–17, 31] and reflects the homogeneity of each statement.

Construct validity is an important element of the validity of a questionnaire. As there were no comparable questionnaires for evaluating back beliefs in our prospective cohort, construct validity was assessed by testing predefined hypotheses about expected correlations to other measurements in our study. The hypotheses were based on existing literature on the BBQ and its assumed relation to similar or non-similar constructs. As expected, a good correlation between the BBQ and the FABQ-PA was found, similar to the original study by Symonds et al., in Britain [5]. Other studies have been conducted on the BBQ and FABQ-PA with populations from different cultures and backgrounds, including Arabic and Chinese low back pain patients [14, 34] and Chinese healthcare students and workers [16, 17]. Their correlation analyses differ from ours and demonstrates low values ranging from − 0.02 [16] to − 0.35 [34]. These results show that healthcare professions and cultural background and origin are important aspects to consider when evaluating back beliefs and fear avoidance behavior due to physical activity. The moderate correlation between the BBQ and RMDQ was also as hypothesized, while the moderate correlation between the BBQ and PCS was slightly lower than expected. Two other studies have investigated the relationship between the Oswestry Disability Index and back beliefs and have found correlation between high disability status and negative back beliefs [33, 40]. To the authors’ knowledge, no previous studies have assessed the relationship between pain catastrophizing (PCS) and back beliefs (BBQ), making this study the first to investigate the correlation between these two scales. The divergent validity was shown by the low correlation found between the BBQ and NRS, and our results are consistent with previous research. Other studies investigating the relationship between the BBQ and pain are finding a low correlation, demonstrating that the degree of pain experienced is not related to pessimistic back beliefs [15, 17, 18, 33]. The exception is that patients with a 1-week history of severe back pain might have more pessimistic back beliefs [31]. The correlation analysis confirmed 75% of the predefined hypotheses, indicating a good construct validity [19].

One limitation of this study is that we could not prohibit participants from seeking medical advice or treatment between recruitment, baseline testing and retesting, and their back beliefs may therefore have been influenced by healthcare or alternative care practitioners. Furthermore, participants were recruited in primary care, and due to practical and economic considerations, there are no data on potential study participants that declined to participate.

## Conclusions

This study indicates that the Norwegian version of the BBQ had acceptable test-retest reliability, internal consistency and construct validity when used on elderly patients in primary care with a new episode of back pain. Further investigations on the importance and consequences of back beliefs are recommended. Research should also explore how to influence irrational attitudes and beliefs about back pain.

## Supplementary information

**Additional file 1.** The Norwegian version of the Back Beliefs Questionnaire.

## Data Availability

The datasets generated and analyzed during the study are available from the corresponding author on reasonable request.

## Abbreviations

BACE: BACk Complaints in Elders
BBQ: Back Beliefs Questionnaire
CI: Confidence interval
FABQ-PA: Fear-Avoidance Beliefs Questionnaire for physical activity
ICC: Intraclass correlation coefficient
LOA: Limits of agreement
MDC: Minimal detectable change
NRS: Numeric Rating Scale
PCS: Pain Catastrophizing Scale
RMDQ: Roland Morris Disability Questionnaire
SD: Standard deviation
SEM: Standard error of measurement
T0: Baseline
T1: Retest
