# Supplementary material for: Back beliefs among elderly seeking health care due to back pain; psychometric properties of the Norwegian version of the back beliefs questionnaire
Source: BMC Musculoskelet Disord. 2019 Nov 3;20:510. doi: 10.1186/s12891-019-2910-8 (PMC6825721; doi:10.1186/s12891-019-2910-8)
Supplement: Supplementary file 1 — Additional file 1. The Norwegian version of the Back Beliefs Questionnaire. [file 12891_2019_2910_MOESM1_ESM.docx]

Appendix 1: The Norwegian version of the Back Beliefs Questionnaire.

**Spørsmål om dine tanker rundt ryggplager**

Vi prøver å finne ut hva folk tenker om problemer i nedre del av ryggen.

Vennligst besvar ALLE påstandene og merk av om du er **enig** eller **uenig** med hvert utsagn ved å sette en ring rundt det tallet som stemmer med det du mener.

| Helt uenig (Disagree) |  | Helt enig (Agree) |
| --- | --- | --- |
| 1 2 | 3 | 4 5 |

| 1 | Det finnes ikke noen effektiv behandling for ryggplager  (There is no real treatment for back trouble) | 1 2 3 4 5 |
| --- | --- | --- |
| 2 | Ryggplager vil på et tidspunkt hindre deg i å være i arbeid  (Back trouble will eventually stop you from working) | 1 2 3 4 5 |
| 3 | Ryggplager betyr perioder med smerter resten av livet  (Back trouble means periods of pain for the rest of one’s life) | 1 2 3 4 5 |
| 4 | Leger kan ikke gjøre noe med ryggplager  (Doctors can not do anything for back trouble) | 1 2 3 4 5 |
| 5 | En dårlig rygg trenger trening  (A bad back should be exercised) | 1 2 3 4 5 |
| 6 | Ryggplager gjør alt i livet verre  (Back trouble makes everything in life worse) | 1 2 3 4 5 |
| 7 | Kirurgi er den mest effektive måten å behandle ryggplager på  (Surgery is the most effective way to treat back trouble) | 1 2 3 4 5 |
| 8 | Ryggplager kan bety at du ender opp i rullestol  (Back trouble may mean you end of in a wheelchair) | 1 2 3 4 5 |
| 9 | Alternativ behandling er den beste behandling for ryggplager  (Alternative treatments are the answer to back trouble) | 1 2 3 4 5 |
| 10 | Ryggplager betyr lange perioder ute av arbeid  (Back trouble means long periods of time off work) | 1 2 3 4 5 |
| 11 | Medisiner er den eneste måten å lindre ryggplager  (Medication is the only way of relieving back trouble) | 1 2 3 4 5 |
| 12 | Når du har hatt ryggplager én gang, vil du alltid være utsatt  (Once you have had back trouble there is always a weakness) | 1 2 3 4 5 |
| 13 | Ryggplager krever hvile  (Back trouble must be rested) | 1 2 3 4 5 |
| 14 | Ryggplager blir gradvis verre med årene  (Later in life back trouble gets progressively worse) | 1 2 3 4 5 |

*Scoring: The ‘inevitability’ measure comprises one scale using a sub-set of 9 statements – items #*

*1,2,3,6,8,10,12,13,14. The score is calculated by reversing the individual scores (ie 5,4,3,2,1) and then summing all 9 statements: High score = better. [The remaining questions (blue) were intended as a ‘treatment’ subscale, but didn’t factor-out consistently in the original (worker) development cohorts. Most researchers have used just the inevitability questions, but the treatment subscale may be worth investigating further in different cohorts].*

Back Beliefs Questionnaire (BBQ) etter Symonds TL et al[. Occup Med (Lond). 1](http://www.ncbi.nlm.nih.gov/pubmed/8672790)996 Feb;46(1):25 -32: Do attitudes and beliefs influence work loss due to low back trouble? Oversatt av Grotle M og Munk R 2014, HiOA.
